# Metabolic and Addiction Indices in Patients on Opioid Agonist Medication-Assisted Treatment: A Comparison of Buprenorphine and Methadone

**DOI:** 10.1038/s41598-020-62556-0

**Published:** 2020-03-27

**Authors:** Igor Elman, Margaret Howard, Jacob T. Borodovsky, David Mysels, David Rott, David Borsook, Mark Albanese

**Affiliations:** 1 Center for Pain and the Brain, Department of Anesthesia, Critical Care and Pain Medicine, Boston Children’s Hospital, Boston, MA USA; 2 Rhode Island Department of Behavioral Healthcare, Cranston, RI USA; 3 Department of Psychiatry, Washington University School of Medicine, St. Louis, MO USA; 4 Department of Psychiatry, Alpert Medical School of Brown University, Providence, RI USA; 5 Department of Cardiology, Sheba Medical Center, Sackler School of Medicine, Tel Aviv, Israel; 6 Center for Pain and the Brain, Department of Anesthesia, Critical Care and Pain Medicine, Boston Children’s Hospital, Massachusetts General Hospital and McLean Hospital, Harvard Medical School, Boston, MA USA; 7 Cambridge Health Alliance, Harvard Medical School, Cambridge, MA USA

**Keywords:** Feeding behaviour, Motivation, Reward, Neuroscience, Diseases, Translational research

## Abstract

Metabolic hormones stabilize brain reward and motivational circuits, whereas excessive opioid consumption counteracts this effect and may impair metabolic function. Here we addressed the role of metabolic processes in the course of the agonist medication-assisted treatment for opioid use disorder (OUD) with buprenorphine or methadone. Plasma lipids, hemoglobin A1C, body composition, the oral glucose tolerance test (oGTT) and the Sweet Taste Test (STT) were measured in buprenorphine- (n = 26) or methadone (n = 32)- treated subjects with OUD. On the whole, the subjects in both groups were overweight or obese and insulin resistant; they displayed similar oGTT and STT performance. As compared to methadone-treated subjects, those on buprenorphine had significantly lower rates of metabolic syndrome (MetS) along with better values of the high-density lipoproteins (HDL). Subjects with- vs. without MetS tended to have greater addiction severity. Correlative analyses revealed that more buprenorphine exposure duration was associated with better HDL and opioid craving values. In contrast, more methadone exposure duration was associated with worse triglycerides-, HDL-, blood pressure-, fasting glucose- and hemoglobin A1C values. Buprenorphine appears to produce beneficial HDL- and craving effects and, contrary to methadone, its role in the metabolic derangements is not obvious. Our data call for further research aimed at understanding the distinctive features of buprenorphine metabolic effects vis-à-vis those of methadone and their potential role in these drugs’ unique therapeutic profiles.

## Introduction

Opioid use disorder (OUD) is an ominous public health problem afflicting about 16 million people worldwide ^1^. In the US, OUD constitutes the seventh cause for disability-adjusted life-years^2–4^; including opioids’ propensity^5^ for the excessive body weight gain (BWG) and its medical sequelae in the form of the ‘Metabolic Syndrome’ (MetS)^5–7^, that is to say, a cluster of interrelated cardiometabolic risk factors comprised of insulin resistance, impaired glucose tolerance, dyslipidemia, abdominal adiposity and hypertension^8–10^. Consequently, even though medication-assisted treatment (MAT) via opioid agonist replacement with long-acting opioids namely, buprenorphine and methadone, usually yields positive clinical outcomes in terms of overdose mortality, infectious diseases, crime, and societal ties^11–14^ concerns have been raised about further worsening of the metabolic status^5^.

There are several lines of evidence that link OUD to metabolic derangements. With regard to genetic antecedents, the TCF7L2 gene codes a transcription factor implicated^15,16^ in non-insulin-dependent diabetes mellitus (NIDDM) and in µ opioid receptor-mediated drug intake^17^. Additionally, melanocortin-4^18^, orexin-1^19^ and OPRM1^20^ genes, involved in appetite and in obesity^21,22^ also underlie opioid consumption^19,23^. Likewise, the appetite-regulating gene^24^ controls the expression of the dopamine D2 receptor gene playing a pivotal role in addiction^21,25^. Biochemically, opioids (e.g., methadone) respectively inhibit and enhance glycolytic- (hexokinase and phosphofructokinase) and gluconeogenesis enzymes (glucose-6-phoaphatase and fructose-1,6-biphosphatase) in the liver and thus produce a state akin to NIDDM^26^. From the digestive perspective, opioids interfere with gastrointestinal motility and with proper food absorption^27^. The endocrine aspects include counterregulatory hormones’ secretion evoked by methadone^28–30^ and alterations in leptin, adiponectin and resistin^ 31^, clinically manifested as NIDDM. Opioids also cause gonadal insufficiency^6,32^, another major contributor to MetS^33^, as well as a reduction in insulin secretion^34–36^ and desensitization of the peripheral insulin receptors^37^.

At the homeostatic level, enhanced µ-opioidergic opioid neurotransmission^38^ respectively boosts and suppresses orexigenic and anorexigenic neuropeptides^39–41^. Opioids likewise enhance hedonic preference for sweet and fatty foods^42,43^ and so are involved in the pathophysiology of food craving and addiction^21,44,45^. Metabolic hormones’ (e.g., insulin) secretion during physiologically-determined anabolism restrains (i.e., increases refractoriness) of the hedonic/motivational neural pathways driving the consumption of both, palatable food^46–48^, and addictive drugs^21^. Caloric deprivation and consequent catabolism conversely predispose for drug seeking and relapse^49^. The above metabolic restraint by insulin is, however, rendered inefficient by sweet and fatty ‘junk’ food^46,47,50^ that is avidly consumed by opioid addicts^43,51,52^ attributable to the exaggerated opioidergic activity enhancing the hedonic appeal of the unhealthy diets^21^. Therefore, a common result in vulnerable individuals could be a feedforward loop whereby high caloric content palatable food produces additional deterioration in the regulatory mechanisms prompting unhealthy eating patterns and opioid consumption to the extent that a *bona fide* MetS, OUD and comorbid conditions^53^ may ensue.

MetS represents a complex pathophysiological condition that may develop in OUD patients^54–57^ even independent of MAT, from increased caloric intake, decreased energy expenditure owing to reduced physical activity or a combination of both^8,21^. This assertion is however undermined by: (1) preponderance of studies reporting low body weight in short half-life opioid (e.g., heroin) abusers who are not on MAT agonist therapy^58–61^ even in the face of glucoregulatory abnormalities^62–64^; and (2) excessive BWG consistently noted in OUD patients following the initiation of methadone^65–71^ and to a lesser degree buprenorphine^6,7,72^ treatment. Nonetheless, the debate about the issue is still ongoing^73^ with a number of reports on the opposite directionality of the metabolic responses^74,75^, including hemoglobin A1C level decreases in buprenorphine-maintained NIDDM patients^76^ in conjunction with heightened insulin sensitivity in methadone-treated OUD patients^77^ as well as hypoglycemia in patients receiving chronic analgesia with methadone^78^. Methadone-induced hypoglycemia was also noted in a rodent model^79^. Paucity of comprehensive metabolic status assessments^5^ or of a proper control adjusting for OUD and for an ongoing opioid agonist therapy^6,69,73^ may partially explain the divergent results. Other potential reasons include the types of opioid receptors engaged^36^, their ligands^79^, the dose^27,80^, acute vs. prolonged opioid exposure^36^ and peripheral^81,82^ vs. central^36,40^ sites of action.

In sum, OUD patients are vulnerable to the development of glucoregulatory alterations that may be worsened by the MAT agonists. Current therapy, focused on dietary caloric restriction, has limited efficacy^83^ and little is known about potential correlates of metabolic dysfunction arising in the context of buprenorphine or methadone treatment. Albeit both agents display a high affinity for the µ receptors, buprenorphine is a partial agonist owing to a distinctive association/dissociation profile contributing to its potentially diminished metabolic side effects vs. methadone that acts as a full opioid agonist^84,85^. Moreover, buprenorphine is an antagonist- whereas methadone is an agonist at the ĸ receptors^84^, a characteristic that may likewise improve the former’s metabolic profile^86,87^. Methadone is further differentiated from buprenorphine by been a non-competitive antagonist at the N-methyl-D-aspartate receptors^88^ that are normatively involved in the suppression of appetite^89,90^, which once again may predispose for the excessive BWG.

Our prior work suggests that blockade of opioid receptors is associated with improved metabolic indices^91,92^ as well as with decreases in rewarding properties of sweet solutions^93^. Here we attempted to extend these findings and to examine the effects of the opposite hyperopioidergic state by employing comprehensive metabolic assessment including plasma lipids, hemoglobin A1C, body composition, the oral glucose tolerance test (oGTT) and the Sweet Taste Test (STT) in OUD patients on buprenorphine- or methadone maintenance. We hypothesized that given the differences in the opioid receptor binding properties, subjects treated with buprenorphine vs. methadone would display more favorable metabolic characteristics and considering the alterations in metabolic restraint on the reward centers^48,94^, subjects with- vs. without MetS would display a worse addiction severity. In an exploratory fashion, potential relationships between the MAT drugs’ exposure duration with metabolic and addiction indices were assessed separately in each study group.

## Methods

### Subjects

All experiments were performed in accordance with relevant guidelines and regulations. Fifty eight subjects participating in MAT with either buprenorphine (n = 26) or methadone (n = 32) were recruited through local advertising and gave written informed consent to the Cambridge Health Alliance (CHA) IRB-approved protocol after the procedures were fully explained. The Diagnostic and Statistical Manual of Mental Disorders, 5th Edition OUD diagnosis was established via the best estimate format using all available sources of information, including history, structured clinical interview with the Mini-International Neuropsychiatric Interview and the Addiction Severity Index (ASI)^95^.

Subjects’ good physical health was determined by history, physical exam and the Cornell Medical Index Health Questionnaire^96^. Weight was measured using a digital electronic scale and height with a Harpenden stadiometer, calibrated on a weekly basis. Body composition (lean and fat body mass) was determined by bioelectric impedance analysis (BIA; RJL Systems, Clinton Township, MI) as described elsewhere by our group^91^. Daily physical activity was self-reported as none = 0, very light = 1, light = 2, moderate = 3, heavy = 4 and elite athlete = 5. Depressive symptomatology was self-rated with the Beck Depression Inventory^97^. The opioid craving questionnaire was modeled after the cocaine craving assessment tool^98^ previously utilized by our group^99^, owing to its ability to predict short-term drug consumption^100^. It measures key aspects of opioid craving (items rated on a scale of 0–10), including (a) current intensity, (b) projected intensity, (c) resistance to opioid consumption, (d) responsiveness to opioid-related conditioned stimuli, and (e) imagined likelihood of opioid consumption if in a setting with access to it. Total spontaneous craving scores were derived by adding together ratings scores on items 1 and 4–6 and subtracting items 2 and 3 resulting in the maximal total score 40.

Subjects were excluded based on pregnancy or a diagnosis of dementia, bipolar disorder, schizophrenia spectrum disorder, major depression, drug/alcohol use disorder (other than OUD), or eating disorder. Also excluded were subjects with potentially confounding medical conditions (e.g., diabetes mellitus, other endocrinopathy, chronic obstructive pulmonary disease, congestive heart failure, hepatitis, hepatic failure, cirrhosis, HIV positive status, end-stage kidney disease, use of opioid antagonists or agonists other than buprenorphine or methadone or use within the past month of drugs with prominent orexigenic or anorexigenic effects e.g., psychostimulants, antihistamines, cannabinoids, dopaminergic or antidopaminergic agents, and mood stabilizers, antidepressants with prominent catecholaminergic effects such as tricylclics, buproprion, mirtazepine, venlafaxine, and duloxetine) or neurological conditions (e.g., seizure disorder, head trauma, past brain surgery, multiple sclerosis, or Parkinson’s disease). Urine toxicology screens were used to confirm MAT adherence and to rule out recent drug (including benzodiazepines) and alcohol consumption; the latter was also ruled out via breathalyzer. The dose was verified by the MAT program and the prescription container label when appropriate.

### Protocol

On the morning of the procedure, subjects reported to the CHA Outpatient Addiction Services, after fasting and refraining from alcohol, tobacco, caffeine, or physical activity for >10 hours. Each of five concentrations of sucrose solution (0.05, 0.10, 0.21, 0.42, and 0.83 M) for STT was presented three times in a pseudorandom order, for a total of 15 samples. Subjects were instructed to sip the solution, swish it around in their mouths, and spit it out. They were then asked to rate “How sweet was the taste?” and “How much do you like the taste?” on a 100-mm analog scale, rinse their mouth with distilled water, and proceed to the next solution.

Glucose is the major energy source for the central nervous system that is neither stored nor produced there. Hence is a need to reward and reinforce behaviors aimed at the procurement of this indispensable fuel that has evolved as the primary reward. oGTT thus has an ecological validity in OUD patients as reward and reinforcement neural pathways is the key etiologic factor not only in food intake, but also in addiction^21,48,101,102^. While in supine position, an intravenous catheter for oGTT blood sampling was placed into the antecubital fossa and kept patent with a slow isotonic (0.9% w/v) saline drip. After resting for 30 minutes, subjects ingested Trutol (Glucose Tolerance Test Beverage) containing 75 g of glucose (Fisher Scientific). Blood samples were collected at 15 minutes before (−15), immediately before glucose ingestion (0), and at 15, 30, 45, 60, 90, and 120 minute time-points. Glucose and insulin plasma concentrations at −15 and 0 minutes were averaged to constitute a single baseline value.

### Biochemical assays

Assays were performed at the CHA Chemistry Laboratory. Plasma glucose concentration was quantified by the hexokinase assay (intra- and inter-assay coefficient of variation 2.00% and 3.00%, respectively). Insulin was measured with the electrochemical luminescence immunoassay (intra-and inter-assay coefficient of variation 1.9% and 2.60%, respectively). Plasma concentrations of total cholesterol (intra-and inter-assay coefficient of variation 2.00% and 3.00%, respectively) and triglycerides (intra-and inter-assay coefficient of variation 2.00% and 2.00%) were measured using enzymatic methods. High- (intra-and inter-assay coefficient of variation 2.3% and 2.7%, respectively) and low (intra-and inter-assay coefficient of variation 2.00% and 4.00%, respectively) density lipoproteins (HDL and LDL, respectively) concentrations were determined via the Siemens Healthineers’ ADVIA Chemistry Systems. High-performance liquid chromatography certified by the Glycohemoglobin Standardization Program was used to measure hemoglobin A1C (intra-and inter-assay coefficient of variation 0.82% and 1.68%, respectively). Insulin resistance and beta-cell function were assessed with the homeostasis model assessment, HOMA-IR and HOMA-β, respectively^103^. Whole-body insulin sensitivity was estimated via the product of the plasma glucose and insulin concentrations during the oGTT quantified as the area under the curve (AUC) using the trapezoid rule^104,105^.

### Statistical analyses

Continuous drug exposure duration was computed as the number of defined daily doses (DDDs) used by an average patient for the MAT indication i.e., 8 mg for buprenorphine and 25 mg for methadone^106^ as the product of daily quantity and duration divided by the DDD for the respective agent^107^. Independent samples t-tests (or Fisher’s exact tests as appropriate) were employed to analyze baseline demographic, clinical, anthropomorphic and biochemical measures’ differences (Table 1). To determine the effects of glucose ingestion on glucose and insulin concentrations, a one-way analysis of variance (ANOVA) with repeated measures design was conducted with the MAT agent (buprenorphine and methadone) as the grouping factor and time (baseline, 15, 30, 45, 60, 90, and 120 minutes) as the within subjects factor. As time-effect was significant, post-hoc Newman – Keuls t-tests were performed to determine if and when changes from baseline were significant.

**Table 1:** Demographic, Clinical, Anthropomorphic and Biochemical Data of Buprenorphine (n = 26)- and Methadone (n = 32)-treated Subjects.

Variable		Buprenorphine	Methadone	Student’s t-test	
				t-value	*p*
Demographic and clinical data	Age (year)	39.92 (9.92)	41.17 (7.92)	−0.53	0.60
	Education (year)	12.54 (1.36)	12.28 (0.99)	0.83	0.41
	ASI (composite score; range 0–1.00)	0.30 (0.14)	0.27 (0.11)	1.00	0.32
	Total craving (score)	−5.13 (13.70)	−6.46 (10.59)	0.40	0.69
	Total BDI (score)	20.52 (16.34)	21.07 (13.98)	−0.13	0.89
	Smoking (cigarette/day)	8.44 (7.87)	9.57 (7.58)	−0.51	0.61
	Daily physical activity (0–5)	1.54 (1.48)	1.78 (1.26)	0.68	0.50
MAT	Dose (mg)	14.85 (4.81)	78.34 (37.99)	n/a	n/a
	Duration (day)	694.80 (809.57)	937.50 (862.98)	−1.08	0.28
	DDD (#)	1272.00 (1445.12)	3036.41 (3560.73)	−2.33	0.02
Anthropometrics	BMI (kg/m^2^)	29.21 (4.36)	30.32 (7.44)	−0.68	0.50
	Body fat mass (kg)	57.77 (17.00)	56.00 (29.42)	0.80	0.26
	Body fat mass (%)	32 (8.10)	29 (11.27)	0.83	0.41
	Fat free mass (kg)	123.33 (15.22)	128.22 (23.63)	−0.87	0.39
	Fat free mass (%)	68.45 (8.10)	70.75 (11.27)	−0.83	0.41
	Total body water (kg)	42.05 (5.13)	43.65 (7.69)	−0.87	0.39
	Total body water (%)	51.45 (6.03)	53.19 (8.57)	−0.83	0.41
Metabolic syndrome	Waist (cm)	101.06 (12.49)	102.98 (5.3)	−0.49	0.62
	Triglycerides (mg/dL)	113.60 (68.72)	133.72 (62.80)	−1.12	0.27
	HDL (mg/dL)	49.39 (15.46)	38.97 (9.98)	3.00	**0.004**
	Systolic BP (mmHg)	119.96 (11.04)	131.25 (21.66)	−2.38	**0.02**
	Diastolic BP (mmHg)	75.15 (9.99)	81.18 (13.53)	−1.85	0.07
	Glucose_baseline_ (mg/dL)	90.78 (13.62)	90.23 (21.78)	0.11	0.91
Metabolic indices	Glucose_120 min_ (mg/dL)	98.56 (28.24)	108.81 (38.95)	−1.07	0.29
	Insulin_baseline_ (mIU/L)	11.67 (9.16)	23.91 (53.02)	−1.23	0.22
	Insulin_120 min_ (mIU/L)	27.82 (22.17)	40.17 (34.16)	−1.52	0.14
	Triglycerides/HDL	2.76 (2.50)	3.87 (2.41)	−1.68	0.10
	Total cholesterol (mg/dL)	173.27 (40.00)	165.04 (42.79)	0.74	0.47
	LDL (mg/dL)	108.42 (35.84)	104.61 (44.11)	0.39	0.70
	Hemoglobin A1C (%)	5.38 (0.44)	5.61 (0.65)	−1.51	0.14
	HOMA-IR (pmol*mmol/L^2^)	2.69 (2.31)	7.10 (17.6)	−1.24	0.22
	HOMA-β (%)	21.93 (704.36)	246.38 (297.98)	−1.47	0.15
	MAP (mmHg)	90.09 (9.04)	97.87 (14.85)	−2.30	**0.03**
	Glucose_AUC_ * Insulin_AUC_	64727891 (39803279)	114114932 (110516922)	−2.10	**0.04**
STT	Taste_0.05 M (mm)	−0.30 (1.32)	−0.25 (1.73)	−0.12	0.91
	Taste_0.1 M (mm)	0.23 (1.04)	−0.12 (1.44)	0.95	0.34
	Taste_0.21 M (mm)	1.47 (1.57)	1.12 (1.42)	0.81	0.42
	Taste_0.42 M (mm)	2.82 (0.98)	2.86 (1.73)	−0.08	0.94
	Taste_0.83 M (mm)	3.88 (1.12)	4.28 (0.87)	−1.36	0.18
	Like_0.05 M (mm)	−0.32 (1.84)	−0.62 (2.36)	0.49	0.62
	Like_0.1 M (mm)	0.00 (1.89)	−0.26 (2.29)	0.33	0.67
	Like_0.21 M (mm)	0.54 (1.61)	0.54 (2.33)	0.39	0.70
	Like_0.42 M (mm)	1.11 (2.01)	0.96 (2.60)	0.20	0.84
	Like_0.83 M (mm)	1.65 (2.25)	1.01 (2.46)	0.70	0.49
				**Fisher’s exact test**	
					*p*
Ratios	Gender (F/M)	13/13	9/23		0.11
	Race (W/B)	24/2	29/3		1.00
	Marital status (S/P)	19/7	29/3		0.10
	Employment (Y/N)	5/21	7/25		1.00
	Metabolic syndrome (Y/N)	5/21	18/14		**0.007**

Data are presented as mean (SD) or ratios.

ASI, Addiction Severity Index; AUC, area under the curve; BMI, Body mass index; DDD, defined daily dose; HDL, High-density lipoprotein; HOMA-IR, homeostasis model assessment-insulin resistance; HOMA-β, homeostasis model assessment-beta cell function; LDL, low-density lipoproteins; Like_0.05 M, lowest sucrose solution concentration liking self-rating; M, Molar; MAP, mean arterial pressure; MAT, Medication-assisted treatment; STT, sweet taste test; Taste_0.05 M, lowest sucrose solution concentration taste self-rating.

General regression model analysis was performed using a model in which the independent variable was the drug exposure duration with the MetS’ components as dependent variables. Both, post-hoc and exploratory correlative analyses (for total cholesterol, LDL, hemoglobin A1C, and opioid craving) were conducted using Pearson product-moment correlation. All analyses were two-tailed with α < 0.05 set as the threshold for statistical significance.

## Results

Table 1 presents demographic, clinical, anthropomorphic and metabolic data for the two groups. Buprenorphine- and methadone-treated patients were not significantly different with respect to age, race, gender, educational, marital and employment status, addiction severity, anthropomorphic measures, physical activity, opioid craving, depressive symptomatology, number of smoked cigarettes, fasting- and 120 min plasma glucose and insulin concentrations, plasma concentrations of total cholesterol, triglycerides, LDL and hemoglobin - A1C. The proportions of subjects with underweight, normal weight, overweight and obesity^108^ was similar (p > 0.41) among buprenorphine (0%, 15.4%, 42.3% and 42.3%) - and methadone (3.1%, 18.8%, 31.2% and 46.9%) - treated groups. Subjects in both groups engaged in very light – light physical activity, and were mildly (buprenorphine: TG/HDL > 2, fasting insulin> 8 uIU/mL and HOMA-IR > 1.5) to moderately (methadone: TG/HDL > 3, fasting insulin> 10 uIU/mL and HOMA-IR > 2.5) insulin-resistant^109^.

Other than the expected solution concentration effect for self-reported taste detection (F = 88.70; p < 0.0001) and for solution liking (F = 5.57; p < 0.003), averaged across the three tasting trials, there were no significant STT group effect or group by concentration interaction (p > 0.44). There were no significant group differences when the proportions (50% vs. 58%) of sweet likers i.e., the ones giving the highest liking rating to the highest sucrose concentration (0.83 M) were compared^110^.

The groups differed with regard to the MAT drug exposure duration (buprenorphine < methadone). Buprenorphine-treated patients presented lower systolic- and mean arterial blood pressure (MAP) and marginally lower diastolic blood pressure in conjunction with higher HDL concentrations and insulin sensitivity index. The group HDL (p = 0.02), but not MAP (p = 0.14) or insulin sensitivity index (p = 0.17) differences remained significant after the adjustment for the drug exposure duration via the analyses of covariance (ANCOVAs). Inclusion of daily physical activity as an additional covariate in the HDL ANCOVA did not significantly alter the group differences’ results (p < 0.02).

MetS was defined in accordance with the American Heart Association/National Heart, Lung and Blood Institute Consensus Statement by three or more of the following criteria^111^: waist circumference >88 cm for women and 102 cm for men, triglycerides ≥150 mg/dL, HDL <40 mg/dL for men and <50 mg/d for women, blood pressure ≥130/85 mm Hg and fasting glucose ≥110 mg/dL^8^. In comparison to the methadone-treated subjects, their buprenorphine-treated counterparts displayed lower rates of MetS (Table 1). Binary logistic regression indicated that there was a significant association between having metabolic syndrome with the study group (Somers’ D = 0.40, p = 0.004), but not with the continuous drug exposure duration (Somers’ D = 0.17, p = 0.13). Subjects with vs. without MetS were maintained on a higher dose of the MAT agent (699.13 ± 484.18 vs. 353.24 ± 330.72; t = 3.12; p = 0.003), though for a similar period of time (28.74 ± 30.98 vs. 27.09 ± 26.75; t = 0.21; p = 0.83). There were no differences (p = 1.0) in the proportion of sweet likers among those with (52%) and without (56%) MetS. There was a trend for a predicted *a priori* heightened ASI composite score in subjects with- vs. without MetS (0.31 ± 0.12 vs. 0.26 ± 0.12; t = 1.72; p = 0.09).

Throughout the 120 minutes following glucose ingestion, both groups demonstrated robust increases (i.e., time effect) in plasma glucose (F = 34.31; p < 0.001) and insulin (F = 12.49; p < 0.001) concentrations. There was a trend for insulin group effect (F = 3.25; p = 0.08), but no glucose group effect or group by time interaction for both biochemicals (p > 0.55). Post-hoc Newman–Keuls tests revealed that plasma concentrations of glucose and insulin significantly increased at 15 minutes (p < 0.002), peaked at 30 minutes (p < 0.0001) and remained significantly elevated at 120 minutes (p < 0.006). Despite excluding participants with previously diagnosed diabetes, two methadone-treated subjects met diabetes criteria based on fasting plasma glucose (FPG) ≥ 126 mg/dL; 120 minutes glucose concentration of one of these subjects was also in the diabetic range i.e., ≥ 200 mg/dL. Three buprenorphine- and three methadone-treated patients had impaired FPG ≥ 110 mg/dL^112^. Two additional subjects in each study group displayed impaired glucose tolerance values based on the 120 minute glucose within the 140–200 mg/dL range.

As shown in Table 2, general regression model analysis revealed that methadone (Wilks’ Lambda = 0.47; F = 6.14; p = 0.0006)-, but not buprenorphine (Wilks’ Lambda = 0.71; F = 1.56; p = 0.22) exposure duration was a significant predictor of MetS. Post-hoc analyses using Pearson product-moment correlation detected that more buprenorphine exposure duration was significantly associated with higher HDL concentration (Table 3); more methadone exposure duration was conversely associated with less HDL-, but higher values of triglycerides, MAP, and fasting glucose (Table 3). Additionally, methadone exposure duration positively correlated with hemoglobin A1C, while neither drug exposure duration correlated with total cholesterol- or LDL plasma concentrations (Table 3). Lastly, buprenorphine-, but not methadone exposure duration inversely correlated with the total opioid craving score. The differences between buprenorphine- and methadone groups’ correlation coefficients^113^ for triglycerides, HDL, MAP, fasting glucose, hemoglobin A1C, and for total craving were statistically significant. There was a trend significance for the MAP correlation coefficients’ differences (Table 3).

**Table 2:** Results of general regression model analysis of the relationship between drug exposure duration and metabolic syndrome components; Wilks’ Lambda = 0.9.71; F = 1.56; p = 0.22 for buprenorphine and Wilks’ Lambda = 0.47; F = 6.14; p = 0.0006 for methadone.

Variable	Buprenorphine			Methadone		
	Multiple R	F	p	Multiple R	F	p
Waist	0.009	0.002	0.97	0.16	0.85	0.36
Triglycerides	0.20	0.99	0.33	0.52	11.46	**0.002**
HDL	0.48	6.98	**0.001**	0.49	9.68	**0.004**
MAP	0.09	0.18	0.68	0.42	6.52	**0.02**
Glucose_baseline_	0.29	2.11	0.16	0.54	12.75	**0.01**

HDL, High-density lipoprotein; MAP, mean arterial pressure.

**Table 3:** Pearson product-moment correlation coefficient between drug exposure duration with metabolic syndrome components, hemoglobin A1C and opioid craving.

Variable	Buprenorphine			Methadone			Comparison	
	r	t	p	r	t	p	z	p
Waist	0.009	0.04	0.97	0.12	0.68	0.50	−0.40	0.35
Triglycerides	−0.20	−0.99	0.33	0.45	2.64	0.01	−2.37	**0.009**
HDL	0.48	2.64	0.01	−0.46	−2.66	0.01	3.56	**<0.0001**
MAP	−0.09	−0.42	0.68	0.38	2.11	0.04	−1.70	**<0.05**
Glucose_baseline_	0.29	1.45	0.16	0.64	4.20	0.0003	−1.57	**0.06**
Total cholesterol	−0.10	−0.51	0.62	−0.04	−0.23	0.82	0.49	0.31
LDL	−0.24	−1.17	0.26	−0.002	−0.01	0.99	−0.79	0.22
Hemoglobin A1C	0.26	1.27	0.22	0.69	4.90	<0.0001	−2.01	**0.02**
Total craving	−0.52	−2.76	0.01	0.15	0.75	0.46	−2.46	**0.007**

HDL, High-density lipoprotein; LDL, Low-density lipoproteins; MAP, mean arterial pressure.

## Discussion

In comparison to buprenorphine-, methadone therapy was associated with heightened rates of MetS, which were also greater (56% vs. 34%) than those in the general populace^114^. The observed MetS rates difference seemed to reflect the respective plasma HDL concentrations and was potentially derived from the unique receptor binding profiles rather than the drugs’ exposure duration. Other group differences such as blood pressure and insulin sensitivity were actually attributable to the drugs’ exposure duration effects. Also, buprenorphine and methadone did not differently affect the anthropometric measurements, addiction-related indices or food preference assessed by means of the STT.

Our data further show that buprenorphine differs from methadone in some of its metabolic correlates. Specifically, buprenorphine exposure duration was positively correlated with the HDL concentrations independently of the physical activity. By contrast, the HDL concentrations were negatively correlated with the methadone exposure duration, which was as well positively correlated with several metabolic indices including triglycerides, MAP, fasting plasma glucose and hemoglobin A1C. An opposite direction of correlations between HDL and drug exposure duration in the study groups suggests that both drugs may be involved in mediating the HDL changes. The correlational nature of the results, however, does not prove the direct MAT-HDL interactions. Thus, it cannot be determined from our results whether HDL concentrations respectively increased or decreased in buprenorphine and methadone-treated subjects as a direct result of the opioid (or other types of) receptors’ engagement or both were a function of lifestyle changes. Further studies exploring potential HDL effects induced by the differential receptors binding profiles characterizing buprenorphine and methadone may help to clarify this issue. Nevertheless, our data are in agreement with a clinical study demonstrating improvements of an HDL-related^114^ metabolic index, namely, hemoglobin A1C^77^, in buprenorphine-treated NIDDM patients.

Significant correlations between methadone drug exposure duration and HDL, MAP, fasting glucose and hemoglobin A1C values are in accord with an earlier study showing that methadone dose was significantly correlated with the odds of having NIDDM^68,69^. Furthermore, our findings extend to the methadone exposure duration the prior report on the predictability of MetS based solely on the exposure time^5^. Although there were methodological similarities between the latter^5^ and the current study (e.g., enrollment of methadone-treated OUD subjects and measures of MetS), there were also important differences, including a sample of buprenorphine-treated patients and the focus on comprehensive clinical, anthropomorphic and biochemical assessments rather than on the MetS *per se*. Thus, this independent replication supports the validity of the relationship between methadone and MetS^9^.

The failure to find a significant relationship between methadone exposure duration and waist circumference, total cholesterol and LDL suggest that methadone may play a less prominent role in the pathophysiological processes affecting cholesterol delivery/turnover or in the preservation of visceral fat evident in the waist circumference^115^. Indeed, there was no significant correlation between waist circumference and total- or LDL cholesterol (p > 0.33); the correlation coefficients indicate that less than 2% of the variance was accounted for by these measures. More research is warranted to address the possibility that each measure captures independent aspects of obesity and of other metabolic abnormalities.

All the same, our data are consistent with the proposition that variations in the methadone exposure adversely affects triglycerides, HDL, blood pressure, insulin sensitivity and MetS as a whole. However, the lack of randomization suggests an additional interpretation that metabolically impaired people actually have a greater severity of opioid addiction^21^ and are thus receiving a higher dose and a longer duration of the methadone therapy. Among correlated factors we cannot determine which are primary and which are secondary using a cross-sectional design. A more complex feedforward interaction is also possible wherein an ongoing opioid consumption brings about metabolic derailments, which further fuel addictive behaviors.

Marginally higher (i.e., trend significance) addiction severity in OUD comorbidity with MetS would support this sort of an interaction regardless of the primary index event. Of note, insulin mediates the homeostatic and reward systems’ cross-talk (impairment of which might underlie the chronically relapsing nature of addictive disorders) and it has been successfully tried in nicotine^116,117^ and cocaine^118^ addiction. It would be of interest to test whether insulin is also able to improve metabolic and reward dysfunction in patients with OUD. Antihyperglycemic compounds as a class along with the long- and short-term anti-obesity drugs may have heuristic value as well. Metformin, for instance, was demonstrated to improve glucoregulatory mechanism via the opioidergic system^119^. Orexin^ 19^ and melanocortin^120^ systems may offer additional exploratory vistas. Such an approach may have merits in patients who are at risk to develop MetS prior to the initiation of agonist MAT (i.e., primary prevention) or who are targeted for an early intervention in the presence of mild metabolic problems (secondary prevention). This proposed strategy underscores the multi-problem nature of OUD and helps easing unnecessary boundaries separating psychiatric and medical OUD formulations, thus fostering inter-disciplinary therapeutic interactions^13^.

A switch from methadone to buprenorphine^121^ is an alternative or complementary strategy. The finding that methadone exposure duration is unrelated to craving suggests a lingering relapse risk during methadone maintenance and emphasizes the significance of its integration with non-pharmacological interventions including contingency management^122^ or mindfulness-based techniques^123^. A potentially beneficial effect of buprenorphine on opioid craving begs further validation. This effect may have pivotal implications for behavioral pharmacology because it could help explicating the currently inexact definition of pharmacodynamic mechanisms inherent in the phenomenology and therapy of opioid craving. Better paradigms for craving research may follow on for the possible benefit of addiction treatment.

The prevalence of overweight and obesity detected in the buprenorphine (84.6%)- and methadone (78.1%)-treated groups was somewhat higher than the numbers (71.6%) reported across the United States^124^. However, while some prior preclinical^125^ and clinical^126,127^ studies support the central opioidergic system involvement in the metabolic side effects of methadone, no such data are available for buprenorphine^128^, and no inferences can be made about buprenorphine’s central mechanisms (or lack thereof) of metabolic changes^6^ from this investigation that was primarily focused on the direct comparison with methadone regarding metabolic and addiction indices. However, before such effects to be investigated, it is first necessary to show that they exist. The latter, and not the former, was the objective of the present study. Its methods may be applied in conjunction with neuroimaging techniques^48^ in future studies in order to address the question of the central mechanisms involvement. Even so, peripheral measures are important because a considerable amount of animal data, where peripheral and central measures were collected simultaneously in the presence of an effective blood-brain barrier^129,130^, suggest that plasma measures may reflect directionally similar changes in the brain^ 131–136^. Therefore changes in plasma biochemicals e.g., glucose and insulin^135–138^ in buprenorphine-treated patients may suggest analogous changes in their brain.

Besides the cross-sectional design and the correlational nature of some of the results, discussed above, this study has additional limitations inherent in the use of the DDD, STT, oGTT and bioelectric impedance. The DDD is a statistically defined unit of measurement that facilitates the comparisons of drugs’ consumption across various geographic locales^106^. It may not necessarily reflect the prevailing prescribing patterns for OUD since 8 mg is half of the target daily buprenorphine dosage^139^, while 25 mg is only about 20% of that for methadone^140^, which could have affected the drug exposure duration comparison. This consideration would not affect the results of the correlative analyses that were performed separately for each study group.

Opioid agonist enhance palatability of sweet food in humans and in laboratory animals^21,93^. This mechanism alone would not explain the clear finding of similar sweet preference in methadone- and buprenorphine-treated subjects and in those with- and without MetS. The STT employed sucrose solutions, but excessive sugar intake is not an exclusive (or even predominant) dietary factor implicated in the development of overweight and obesity when compared with saturated fat^141,142^. Although a more realistic stimulus for this study could have been some type of highly palatable mix of macronutirents (e.g., a milkshake)^143^, we believe that it would be most useful to first subdivide the process of food reward into basic subtypes. Each subtype can be then studied separately, thus providing a sound footing for understanding a potential interaction between different macronutrients and their role in the generalized rewarding nature of food and metabolic derailments.

Two participants were diagnosed with diabetes and more had impaired glucose tolerance based on the oGTT. FPG, the most common tool for monitoring metabolic status, is predominantly a measure of hepatic glucose production in a fasting state. Though more time-consuming and costlier than FPG, oGTT measures glucose and insulin sensitivity related to food intake, which is more ecologically valid considering that patients spend relatively little time in a fasting state. Other measures of insulin sensitivity and secretion (e.g., euglycemic and hyperglycemic clamp) might provide greater flexibility for evaluation of associated abnormalities in intermediary metabolism. The Frequently Sampled Intravenous GTT is probably the most efficient method for obtaining both measures with sufficient precision to guide the direction for future studies in this area. Besides, measuring of visceral fat (abdominal magnetic resonance imaging)^35^ in addition to total body fat (bioelectric impedance) would enable to determine whether substantial differences exist that should be pursued in more detail.

In conclusion, the presented results suggest that two MAT agonists, buprenorphine and methadone, share similarities, but have important differences with regard to their metabolic profiles. Both drugs are associated with overweight, obesity and insulin resistance. Owing, in part, to better HDL values buprenorphine, as compared with methadone, produces lower rates of MetS, which in turn tends to be accompanied by a greater addiction severity. Buprenorphine exposure duration may be associated with better HDL and opioid craving values. Conversely, methadone-, but not buprenorphine exposure duration may be involved in the triglycerides-, HDL-, blood pressure-, fasting glucose- and hemoglobin A1C adverse effects.

Given the morbidity and mortality related to OUD, and the data demonstrating the efficacy of MAT, buprenorphine and methadone will remain as crucial interventions. The process of administering MAT may, however, need to be adjusted. For example, potential for metabolic derangements should be included in the informed consent discussion prior to implementation of a treatment plan. Our findings further underscore the importance of the focus on lifestyle issues such as diet and exercise. Given that buprenorphine is associated with fewer metabolic irregularities and a beneficial craving effect, it should be considered the default first step if the agonist treatment is considered. Finally, future studies are needed for elucidation the mechanism of MAT and its central nervous system sequelae along with optimal metabolic screening and monitoring standards of MAT patients.
