# Author Correction: Metabolic and Addiction Indices in Patients on Opioid Agonist Medication-Assisted Treatment: A Comparison of Buprenorphine and Methadone

**DOI:** 10.1038/s41598-020-64610-3

**Published:** 2020-05-01

**Authors:** Igor Elman, Margaret Howard, Jacob T. Borodovsky, David Mysels, David Rott, David Borsook, Mark Albanese

**Affiliations:** 1 Center for Pain and the Brain, Department of Anesthesia, Critical Care and Pain Medicine, Boston Children’s Hospital, Boston, MA USA; 2 Rhode Island Department of Behavioral Healthcare, Cranston, RI USA; 3 Department of Psychiatry, Washington University School of Medicine, St. Louis, MO USA; 4 Department of Psychiatry, Alpert Medical School of Brown University, Providence, RI USA; 5 Department of Cardiology, Sheba Medical Center, Sackler School of Medicine, Tel Aviv, Israel; 6 Center for Pain and the Brain, Department of Anesthesia, Critical Care and Pain Medicine, Boston Children’s Hospital, Massachusetts General Hospital and McLean Hospital, Harvard Medical School, Boston, MA USA; 7 Cambridge Health Alliance, Harvard Medical School, Cambridge, MA USA

Correction to: *Scientific Reports* 10.1038/s41598-020-62556-0, published online 27 March 2020

The Acknowledgements section in this Article was omitted. The Acknowledgements section should read:

“The authors would like to acknowledge the Clinical Team at the Outpatient Addiction Services, Cambridge Health Alliance for dedicated clinical support provided on this study and Delany Berry (Center for Pain and the Brain, Boston Children's Hospital) for her assistance with editing and formatting this manuscript.”

